# De novo transcriptome analysis and identification of defensive genes in garlic (*Allium sativum* L.) using high-throughput sequencing

**DOI:** 10.1186/s43141-023-00499-5

**Published:** 2023-05-10

**Authors:** Malyaj R. Prajapati, Jitender Singh, Pankaj Kumar, Rekha Dixit

**Affiliations:** 1 Division of Microbial and Environmental Biotechnology, College of Biotechnology, Sardar Vallabhbhai Patel University of Agriculture and Technology, Meerut, Uttar Pradesh 250110 India; 2 Department of Microbiology, Chaudhary Charan Singh University, Meerut, Uttar Pradesh 250004 India

**Keywords:** *Allium sativum* L. Defensive gene, Illumina sequencing, KEGG

## Abstract

**Background:**

Garlic (*Allium sativum L.*) is the second most widely cultivated Allium which is mainly grown in temperate regions and used as a flavoring agent in a wide variety of foods. Garlic contains various bioactive compounds whose metabolic pathways, plant–pathogen interactions, defensive genes, identify interaction networks, and functional genomics were not previously predicted in the garlic at the genomic level. To address this issue, we constructed two garlic Illumina 2000 libraries from tissues of garlic clove and leaf.

**Results:**

Approximately 43 million 125 bp paired-end reads were obtained in the two libraries. A total of 239,973 contigs were generated by de novo assembly of both samples and were compared with the sequences in the NCBI non-redundant protein database (Nr). In total, 42% of contigs were matched to known proteins in public databases including Nr, Gene Ontology (GO), and Cluster Orthologous Gene Database (COG), and then, contigs were mapped to 138 via functional annotation against the Kyoto Encyclopedia of Genes and Genomes pathway database (KEGG). In addition, a number of regulatory genes including the CCHC (Zn) family, followed by WD40, bromodomain, bZIP, AP2-EREBP, BED-type (Zn) proteins, and defense response proteins related to different conserved domains, such as RGA3, NBS-LRR, TIR-NBS-LRR, LRR, NBS-ARC, and CC-NBS-LRR were discovered based on the transcriptome dataset. We compared the ortholog gene family of the *A. sativum* transcriptome to *A. thaliana*, *O. sativa*, and *Z. mays* and found that 12,077 orthologous gene families are specific to *A. sativum* L. Furthermore, we identified genes involved in plant defense mechanisms, their protein–protein interaction network, and plant–pathogen interaction pathways.

**Conclusions:**

Our study contains an extensive sequencing and functional gene-annotation analysis of *A. sativum* L. The findings provide insights into the molecular basis of TFs, defensive genes, and a reference for future studies on the genetics and breeding of *A. sativum* L.

## Introduction

Garlic (*Allium sativum*) is one of the most vital remunerative bulbous spices and medicinal crops grown commercially. Since 1970, world garlic production has increased more than 10 times, while the cultivated area has increased approximately four times, indicating an improvement in yield. In addition to fresh consumption, the production of dried and processed garlic products is used in the food industry as dietary health food supplements and in the treatment of many diseases [31]. In garlic, the major flavor precursor is sulfur-alk(en)yl cysteine sulfoxide (ACSO, alliin), which is degraded by alliinase enzymes upon tissue disruption to give pyruvate, ammonia, and thiosulfinate. These products are the source of the very characteristic aroma of garlic and the proposed health-beneficial properties [19, 34].

The nuclear genomes of many *Allium* species are specifically large among eukaryotes: the 2C DNA quantity per genome in *Allium* species ranges from 16.93 to 63.57 pg. Garlic is a diploid (2n = 2 ×  = sixteen) plant with a nuclear genome of 15,901 Mbp consistent with 1C, slightly smaller than onion and thirty-two and 6 times larger than rice and maize, respectively [4]. Due to its massive genome, few genomic statistics are available in *Allium* species. In one study, 11,008 ESTs derived from a cDNA library of onions were sequenced [1]. In garlic, the next-generation sequencing analysis based on RNA from renewal buds resulted in de novo assembly of 128,000 unigenes that were annotated and analyzed with respect to Gene Ontology (GO) and metabolic pathways [28]. In addition, 352 differentially expressed transcript-derived fragments showed differential expression in the leaf, meristematic, and flower tissues [6].

In recent years, the development of next-generation sequencing (NGS) technology has offered a significant and cost-efficient tool for the generation of transcriptomic datasets in nonmodel species using several platforms, such as Roche 454, Illumina HiSeq, and Applied Biosystems SOLiD [2]. RNA sequencing has been used for the genome-wide quantification of absolute transcript levels, the identification of novel genes, the characterization of transcript structure (including 50 and 30 ends, introns, and exons), and the mining of molecular markers. Various nonmodel organisms, such as *Allium* cepa, Jerusalem artichoke, Sophora japonica, and Youngia japonica, have been studied by next-generation sequencing, which has offered a better understanding of these plants [7–9]. In the present study, high-throughput sequencing data was used to analyze the transcriptome of *Allium sativum* L. cloves and leaves. The defensive genes involved in metabolic pathways and their regulation and protein–protein interaction networks were identified. Additionally, the SSR markers developed here should facilitate marker-assisted selective breeding for elite germplasm, gene mapping, and linkage map development in *Allium sativum* L.

## Materials and methods

### Plant materials and RNA extraction

Garlic (*Allium sativum* L.) cloves and leaves were collected in Horticulture Research Center, Sardar Vallabhbhai Patel University of Agriculture and Technology, Meerut, Uttar Pradesh, India, during March 2018. The sample tissues were immediately frozen in liquid nitrogen and stored at − 80 °C until use. The total RNA was isolated with a GeneJET RNA Purification Kit (Thermo Scientific) according to the manufacturer’s instructions. RNA quality was verified using a UV spectrophotometer (UV-1800, Shimadzu).

### cDNA library construction and high‑throughput RNA sequencing

Two RNA-Seq libraries were constructed separately using 200 ng of the total RNA from the pooled tissues of garlic cloves and leave samples. Poly-A-containing mRNAs were purified from the total RNA samples using the OligoTex mRNA mini kit (Qiagen, Germany). The mRNA was then fragmented into small pieces using an RNA fragmentation reagent (Ambion™, Life Technologies Corporation). Using these short fragments as the templates, the first cDNA strand was synthesized using random hexamer primers and reverse transcriptase (Thermo Scientific), and the second-strand cDNA was synthesized using DNA polymerase I and RNase H. The cDNA fragments were purified using the QiaQuick PCR extraction kit (Qiagen, Germany) and resolved with EB buffer for end reparation and poly(A) addition. The short fragments were then connected with sequencing adapters, and the products were subsequently purified and amplified via PCR to create the final cDNA libraries. The cDNA library was sequenced using an Illumina HiSeq 2000 at NxGenBio Life Sciences, New Delhi.

### Data filtering and de novo transcripts assembly

The quality of the sequencing data was checked, and preprocessing of the data was performed using CLC Workbench 7.0.4 software. Low-quality reads with a Phred score ≤ 30 and reads containing ambiguous bases “N” were removed. Adaptor sequences with low-quality bases from 5′ and 3′ were trimmed to improve the quality of the data. De novo assembly of the filtered clean data was performed using CLC Workbench 7.0.4 software, and quality control (QC) was performed.

### Functional annotation and pathway assignments

Assembled garlic contigs were used as queries in the Blastx algorithm using OmicsBox 1.2 (https://www.biobam.com/omicsbox) software against the nonredundant (NR) databases at NCBI. The resulting blast hit with an *e* value of ≤ 1.0E − 3 was considered a significant match for further functional annotation of contigs. BLASTX alignments between unigenes and protein databases, including NR, Kyto Encyclopedia Gene and Genome Database (KEGG), and cluster orthologous gene database (COG) were performed. Protein coding sequences were characterized by an InterPro domain search directly on the FASTA input file of the contigs, and Gene Ontology terms were assigned to the identified domains. InterPro Scan via Omicsbox 1.2 (https://www.biobam.com/omicsbox) was queried against nine databases: BlastProDom, FPrintScan, HMMPIR, HMMPfam, HMMSmart HMMTigr, Profile Scan, ScanRegExp, and SuperFamily. Functional information for each contig was retrieved from the Gene Ontology (GO) database encapsulating millions of functionally annotated gene products for several different species. Moreover, the GO database contains an evidence code qualifier that provides information related to the quality of this functional assignment. Omicsbox 1.2 annotation was performed with default parameters after Gene Ontology mapping, which enumerated the GO annotation score for each candidate GO term. The Cluster of Orthologous Groups database (COG) annotation was performed using the BLASTX algorithm against the COG. The functional annotation by Gene Ontology terms was performed using the Omicsbox 1.2 program (https://www.biobam.com/omicsbox). The Kyoto Encyclopedia of Genes and Genomes (KEGG) database pathway annotation was performed by sequence comparisons against the KEGG database using BLASTX with an *e* value threshold of 1.0E − 3.

### Identification of defensive genes in the garlic transcriptome

To explore the defensive genes/disease resistance genes for each transcript against various environmental stresses, fungal, bacterial, and other infections on garlic, annotation was carried out. To validate the results, all contigs were queried against the databases of *Arabidopsis thaliana*, *Zea mays*, and *Oryza sativa* via the OmicsBox 1.2 suite mapping and annotation suite.

### Comparative analysis of multispecies orthologous gene families and detection of SSR markers

Multispecies genome comparisons and visualization of orthologous clusters between *Allium sativum* L., *Arabidopsis thaliana*, *Oryza sativa*, and *Zea mays* were performed using the online web server OrthoVenn2. (https://orthovenn2.bioinfotoolkits.net/) [32]. The mining of simple sequence repeat (SSR) markers was performed using the MISA (microsatellites identification tool) Perl script [5], which identified both perfect and compound repeats. We searched for SSRs with motifs ranging from di to hexanucleotides in size. The parameters were adjusted for the identification of perfect di, tri, tetra, penta, and hexanucleotide motifs with a minimum of 9, 6, 5, 4, and 3 repeats, respectively. Adjacent microsatellites ≤ 10 nt apart were considered compound repeats [12].

## Results

### High-throughput sequencing and assembly

To obtain a comprehensive overview of *Allium sativum*, RNA was isolated from two tissue samples and sequenced on the Illumina HiSeq 2000 platform (125 bp paired-end). A total of 43 million raw read fragments were achieved in the two libraries. Raw reads were explored for the overall relative 43.8% GC content found in all the reads. A total of 34,873,376 and 31,497,569 sequences were trimmed with an average length of 124.54, respectively, garlic cloves and leaves and the post-trimming results are summarized in Table 1. All raw sequence data have been deposited in the NCBI Sequence Read Archive (http://www.ncbi.nlm.nih.gov/Traces/sra/) under the accession number SRX8876914 and SRX8862808. De novo assembly of processed garlic clove reads was carried out to generate contig sequences with a CLC genomics workbench, and contigs were formed in two groups, including scaffolded regions and excluding scaffolded regions. Garlic transcripts, including scaffolded regions, were taken into account for further annotation and analysis. A bulk of 131,305 and 1,08,668 contigs were generated, respectively from garlic cloves and leaves. Therefore, contigs were transferred to the OmicsBox 1.2 suite for further mapping and annotation.

**Table 1:** Adapter trimming report of paired-end sequenced dataset of garlic transcriptome

Sample	Input reads	Average length	No. of read after trimming	Percentage trimmed (%)	Average length after trim
**Clove**	34,873,376	125.0	34,873,264	100.0	124.54
Leaf	31,497,569	124.99	31,494,032	99.99	124.57

### Functional annotation and classification of the garlic transcriptome

**Fig. 1:**
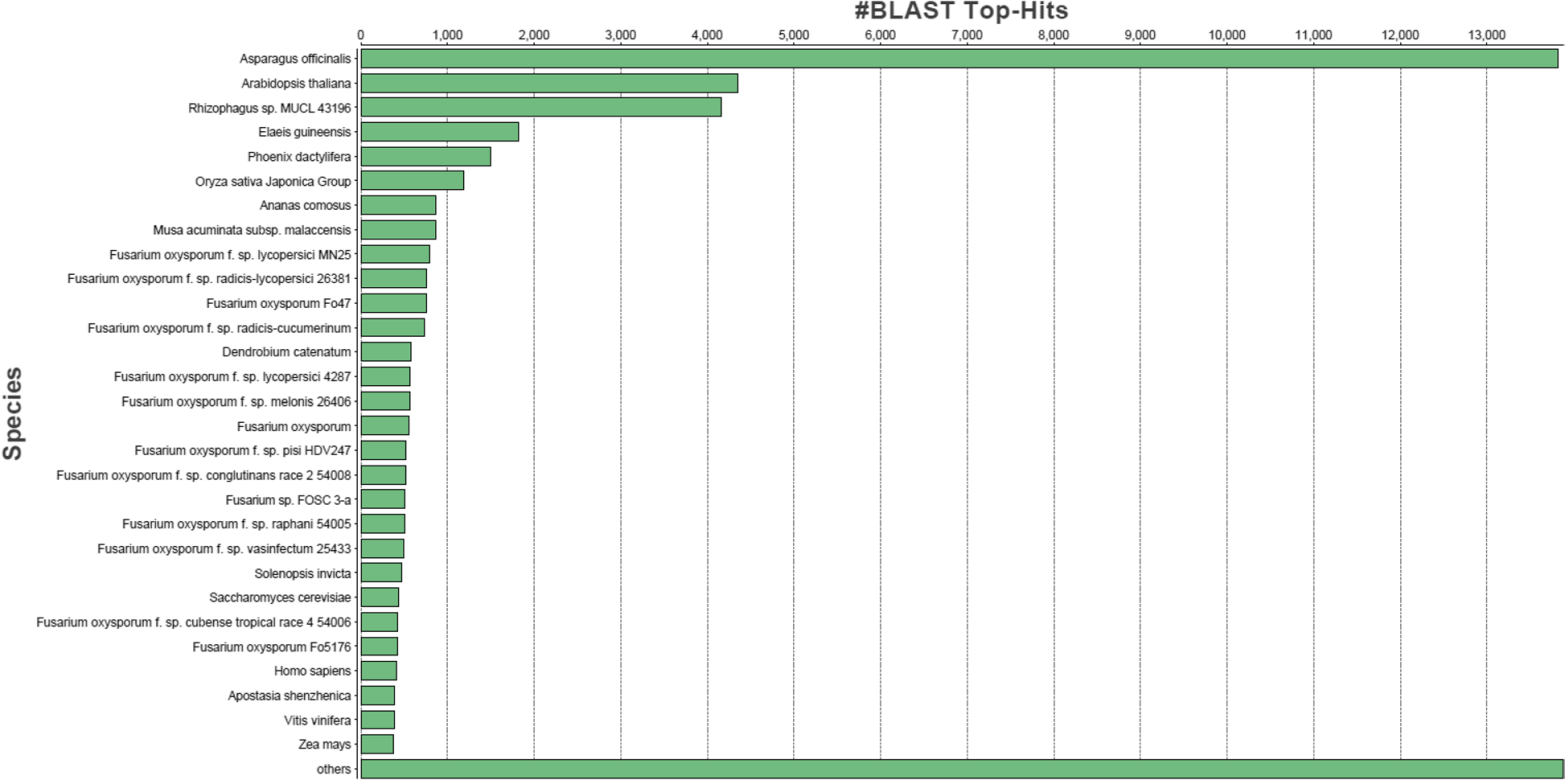
Blast analysis showing similarity between garlic transcriptomic contigs and NR database

**Fig. 2:**
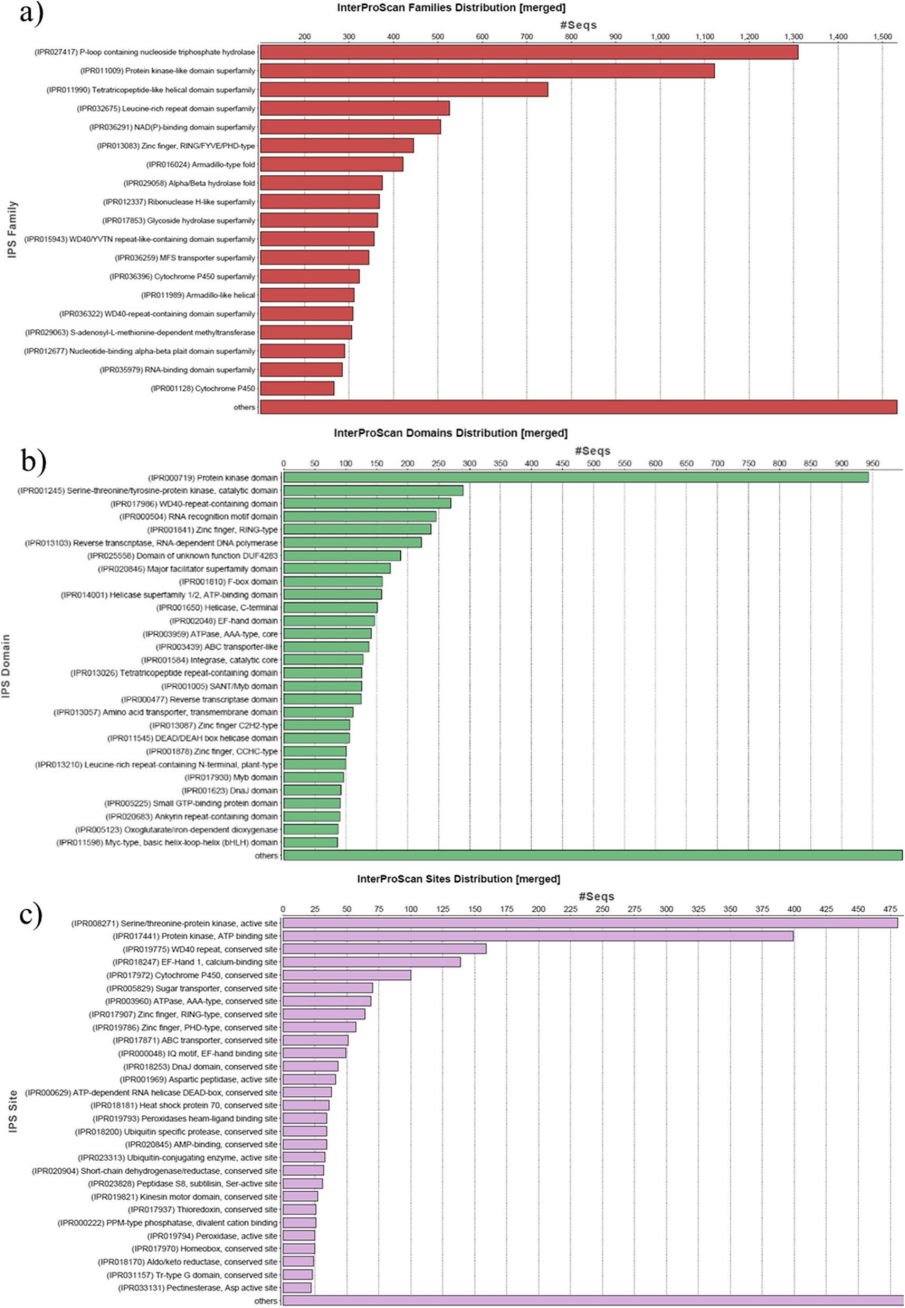
Graph depicts the search results for garlic transcripts via interpro scan using OmicsBox 1.2 suite: **a** Distribution of putative transcripts among the protein families. **b** Major protein domains and their respective distribution among contigs. **c** Repeated regions found in garlic transcript sequences

Annotation of contigs was achieved based on searches of specific databases for sequence similarity using OmicsBox 1.2 suite. All of the contigs were compared to the sequences in the NCBI (http://www.ncbi.nlm.nih.gov/) nonredundant protein (nr) database using BLASTX with a cutoff *e* value of 10 − 3. Out of 131,305 garlic contigs, 80,755 contigs showing no significant blast hit against NR db v5. Species expressing considerable similarity with garlic contig sequences are shown in Fig. 1 against NR db v5. Asparagus officinalis, *Arabidopsis thaliana*, Rhizophagus sp., and Oryza sativa japonica groups showed dominant blast hits against NR db. Interpro scan results were explored for protein families, domains, and repeats. Protein families distributed among contigs with identifiers include P-loop containing nucleoside triphosphate hydrolase (IPR027417) at the highest peak followed by protein kinase-like domain (IPR011009), tetratricopeptide-like helical domain superfamily (IPR011990), leucine-rich repeat domain superfamily (IPR032675), NAD(P)-binding domain superfamily (IPR036291), zinc finger (IPR013083), RING/FYVE/PHD-type, Armadillo-type fold (IPR016024), Alpha/Beta hydrolase fold (IPR029058), ribonuclease H-like superfamily (IPR012337), glycoside hydrolase superfamily (IPR017853), WD40/YVTN repeat-like superfamily (IPR036322), MFS transporter superfamily (IPR036259), cytochrome P450 superfamily (IPR036396), WD40-containing repeat-containing domain superfamily (IPR036322), and MFS transporter (IPR0379). Many contigs represent important domains were represented by like as heat shock protein (Hsp90) (IPR001404), N-terminal domain (IPR020575), DNA mismatch repair protein MutL/Mlh/Pms (IPR038973), trigger factor, ribosome-binding, and bacterial protein (IPR008881) (Fig. 2a). Protein domains identified among transcripts were Cytochrome P450 (IPR017972), followed by zinc finger, RING-type (IPR001841) RNA recognition motif domain (IPR000504), small GTPase superfamily (IPR001806), reverse transcriptase (IPR013103), Helicase superfamily 1/2, ATP-binding domain (IPR014001), SANT/Myb domain (IPR001005), and DnaJ domain and (IPR001623), etc. (Fig. 2b). Repeated regions were captured as they are formed to play vital roles in biological processes. The graphical distributions of the major identified repeats include WD40 repeat (IPR001680), tetratricopeptide repeat-containing domain (IPR013026), leucine-rich repeat-containing N-terminal (IPR013210), pentacotripeptide-repeat region of PRORP (IPR033443), TAF6, C-terminal HEAT repeat domain (IPR011442) and VPS13, and repeated coiled region (IPR031642 (Fig. 2c).

**Fig. 3:**
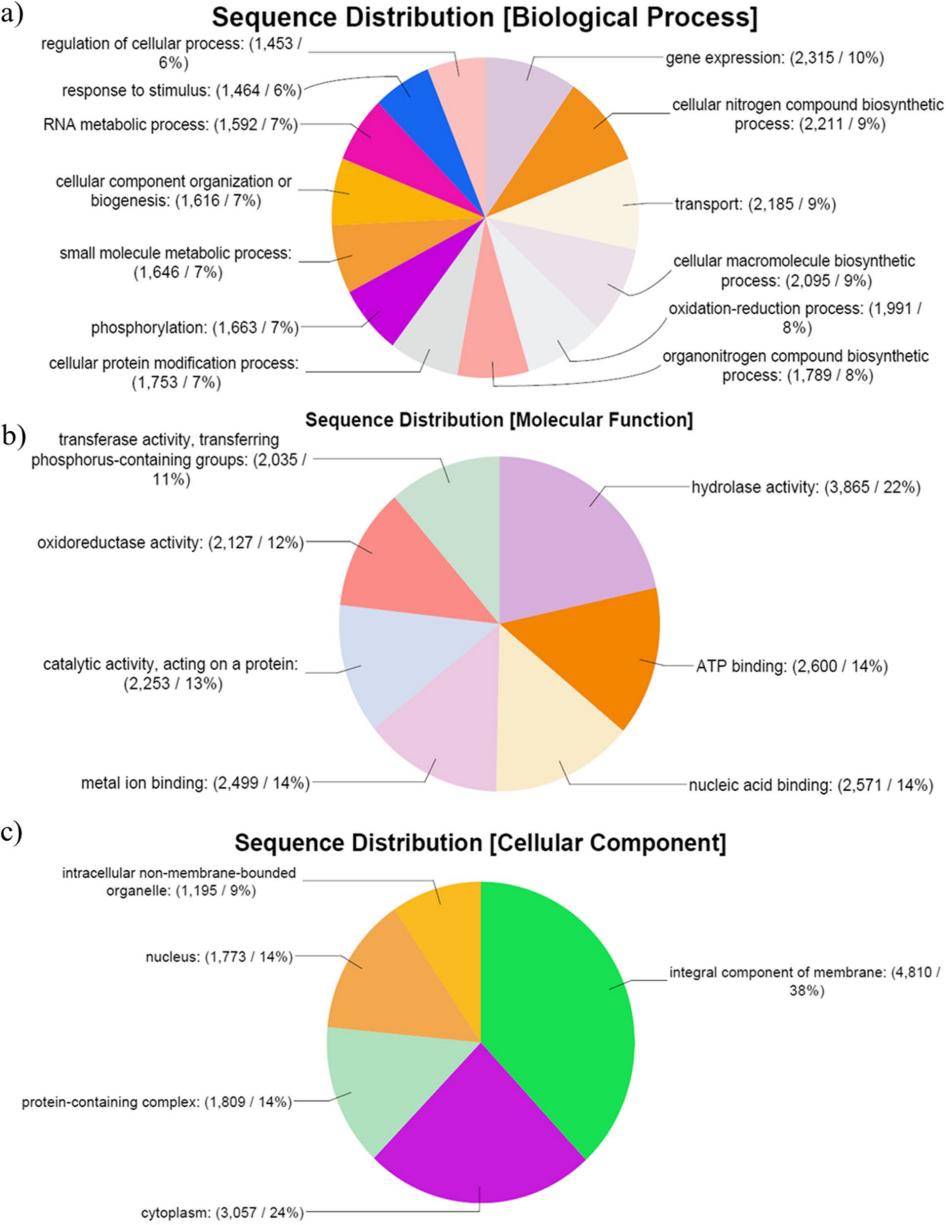
Functional classification of garlic contigs based on Gene Ontology (GO): **a** biological function, **b** molecular function, and **c** cellular components

**Fig. 4:**
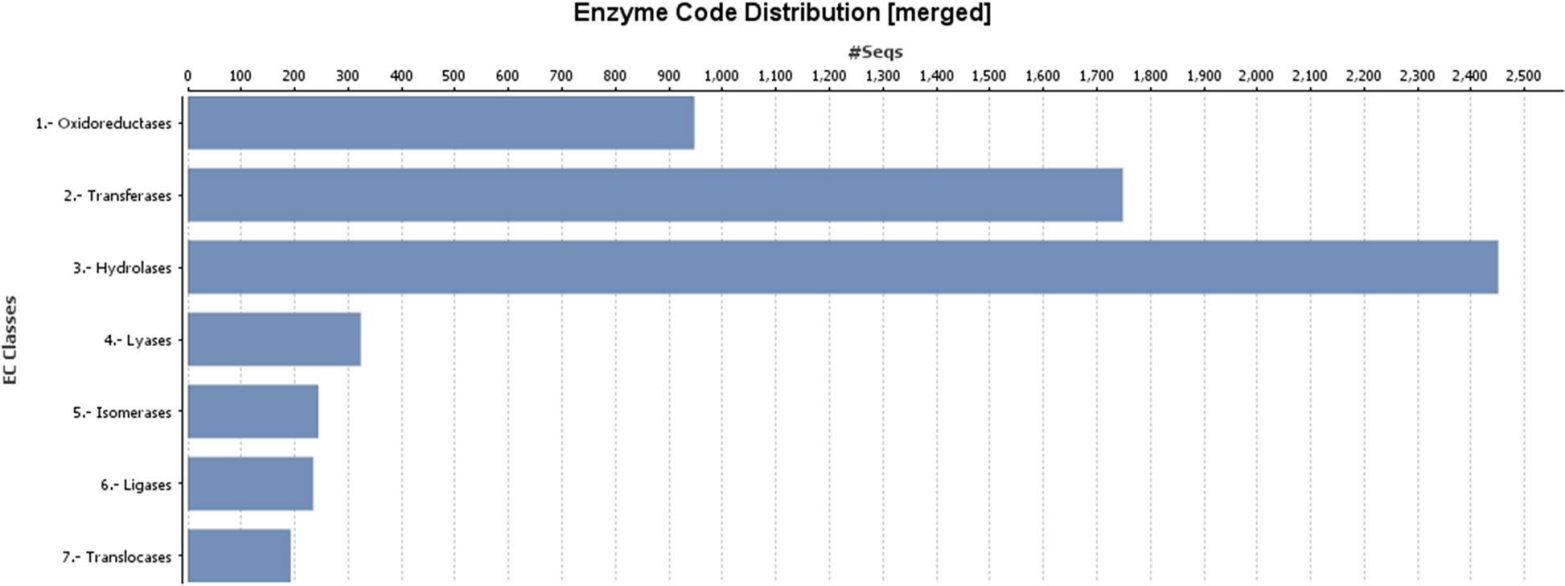
Distribution of enzyme classes and the numbers of contigs laying in these classes

In mapping and annotation, the latest version of the GO db search provides evidence code qualifiers with each retrieved GO term, which suggests the quality of the functional assignment of each assembled garlic contig. Evidence code distribution with garlic contigs as well as with their blast hits is summarized graphically. Gene Ontology mapping and annotation are shown in three categories associated with putative transcripts viz. biological process, cellular component, and molecular function. Out of the three main categories, contigs associated with biological processes were found to be dominant, followed by contigs associated with molecular functions and cellular components. The biological processes were classified into subcategories. Among them, the maximum number of contigs was found to be associated with metabolic processes (10,592; 37%), followed by cellular processes (9461; 33%), localization (2239; 8%), response to stimuli (1464; 5%), and regulatory biological processes (1650; 6%) (Fig. 3a). Furthermore, few putative garlic transcripts were assigned to subcategories such as developmental and multicellular organismal processes. The transcripts engaged with molecular functions were found to be associated with catalytic activity (2253; 13%) and binding function (32,004; 71%) (Fig. 3b). On the other hand, cellular components included 5 subcategories with 23,585 contigs. The most represented subcategory was membrane (6749 contigs; 29%), followed by intrinsic components of membrane (4830; 69.9%), organelle (4550; 19%), intracellular organelle (4399; 19%), and cytoplasm (3057; 13%) (Fig. 3c). Additionally, considering enzyme (coded by contigs) code distribution among enzyme classes, the highest number of contig sequences was found to have the genes that code for hydrolytic enzymes (Fig. 4).

### KEGG pathway annotation

**Fig. 5:**
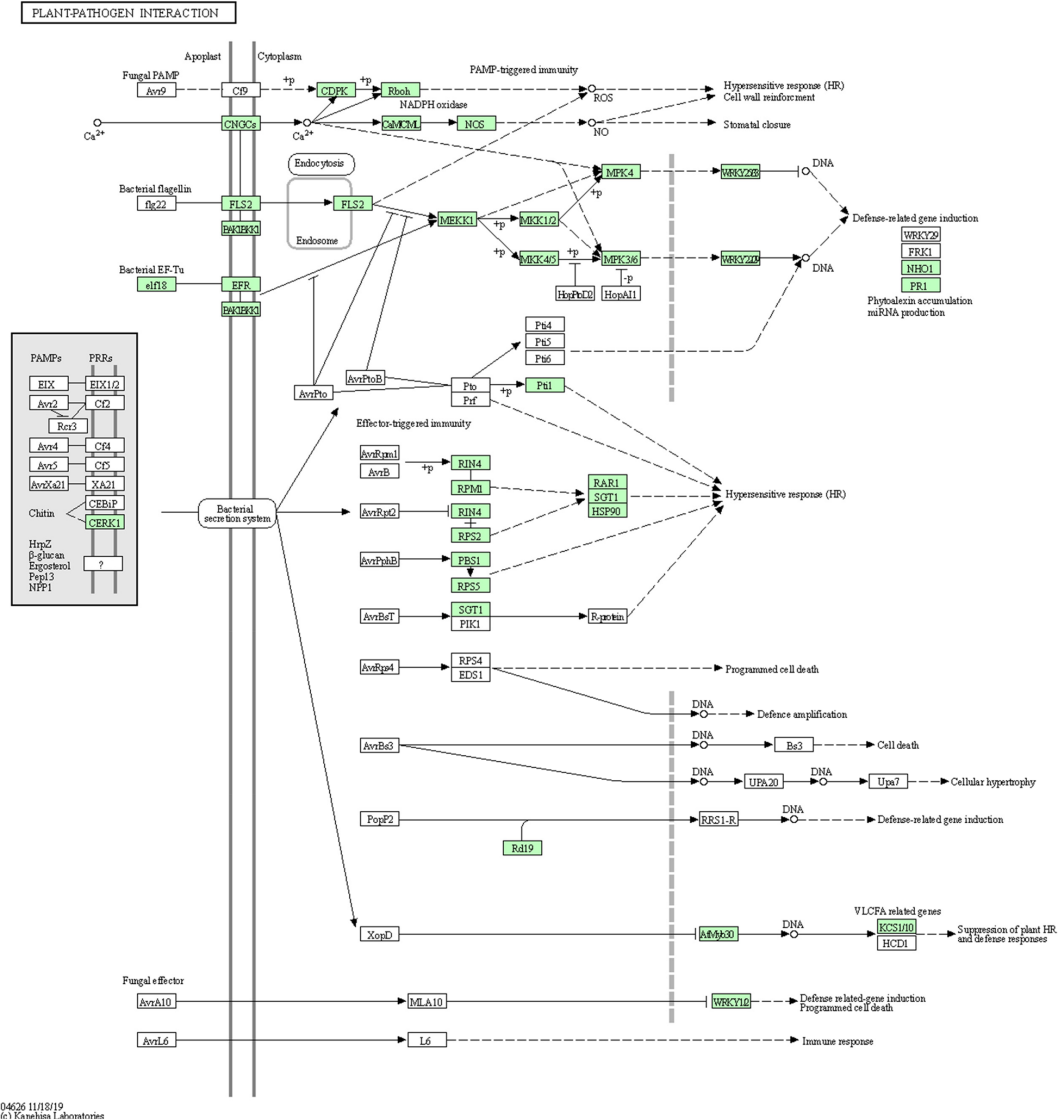
Plant–pathogen interaction pathway. Thirty-five contigs were assigned to plant–pathogen interaction pathway generated by KEGG

Putative transcripts were screened against the KEGG database to analyze the gene products of metabolic processes and related cellular processes, which resulted in 138 KEGG pathways assigned to garlic transcripts. Primarily among 138 pathways, the most represented pathway by contigs were related to biosynthesis of antibiotics (468) and then metabolism which includes Purine metabolism (167); Thiamine metabolism (168); Cysteine and Methionine metabolism (35); Amino sugar and nucleotide sugar metabolism (128); Glycine, serine, and threonine metabolism (29); Pyruvate metabolism (28); Porphyrin and chlorophyll metabolism (26); starch and sucrose metabolism (25); glycolysis/gluconeogenesis (202); oxidative phosphorylation (146); Pyrimidine metabolism (67); drug metabolism—cytochrome P450 (57); plant–pathogen interaction pathway (35) (Fig. 5); glyoxylate and dicarboxylate metabolism (22); glycerophospholipid metabolism (67); sulfur metabolism (16); propanoate metabolism (14); nitrogen metabolism (25); tyrosine metabolism (69); and metabolism of Xenobiotics by cytochrome P450 (55). In contrast, only a few putative transcripts were associated with biotin metabolism (11), vitamin B6 metabolism (6), retinol metabolism (30), caffeine metabolism (4), styrene degradation (4), xylene degradation (1), steroid degradation (2), atrazine degradation (1), photosynthesis (1), and beta-lactam resistance (1). Many garlic contigs were classified into pathways related to the biosynthesis of secondary metabolites, such as biosynthesis of antibiotics (147), terpenoid backbone biosynthesis (22), flavonoid biosynthesis (17), zeatin biosynthesis (9), streptomycin biosynthesis (5), steroid biosynthesis (4), novobiocin biosynthesis (5), carotenoid biosynthesis (4), isoquinoline alkaloid biosynthesis (4), indole alkaloid biosynthesis (2), diterpenoid biosynthesis (2), monoterpenoid biosynthesis (1), sesquiterpenoid and triterpenoid biosynthesis (1), biosynthesis of vancomycin group antibiotics (10), aflatoxin biosynthesis (1), and biosynthesis of Ansamycins (1).

### Transcripts related to transcription factors

**Fig. 6:**
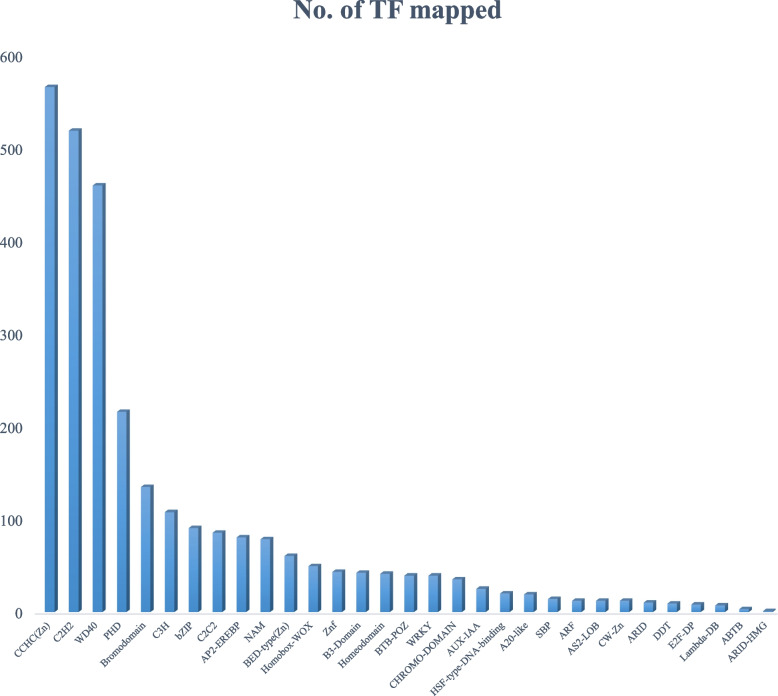
Distribution of TF family and the numbers of contigs laying in these family

Transcription factors (TFs) regulate gene expression patterns, which in turn determine several biological processes. Out of 130,935 contigs, 3687 contig plant-specific and plant-nonspecific transcription factor families were observed (Fig. 6). Most of them represent the CCHC(Zn) family, followed by WD40, bromodomain, bZIP, AP2-EREBP, BED-type (Zn), etc. In addition to the identified transcript, 30.93% belonged to the category of the plant-specific transcription factor family. Most of them represent the AP2EREBP family, followed by WRKY and AUX-IAA.

### Identification of *A. sativum* defense-responsive genes based on the de novo assembled transcriptome

**Fig. 7:**
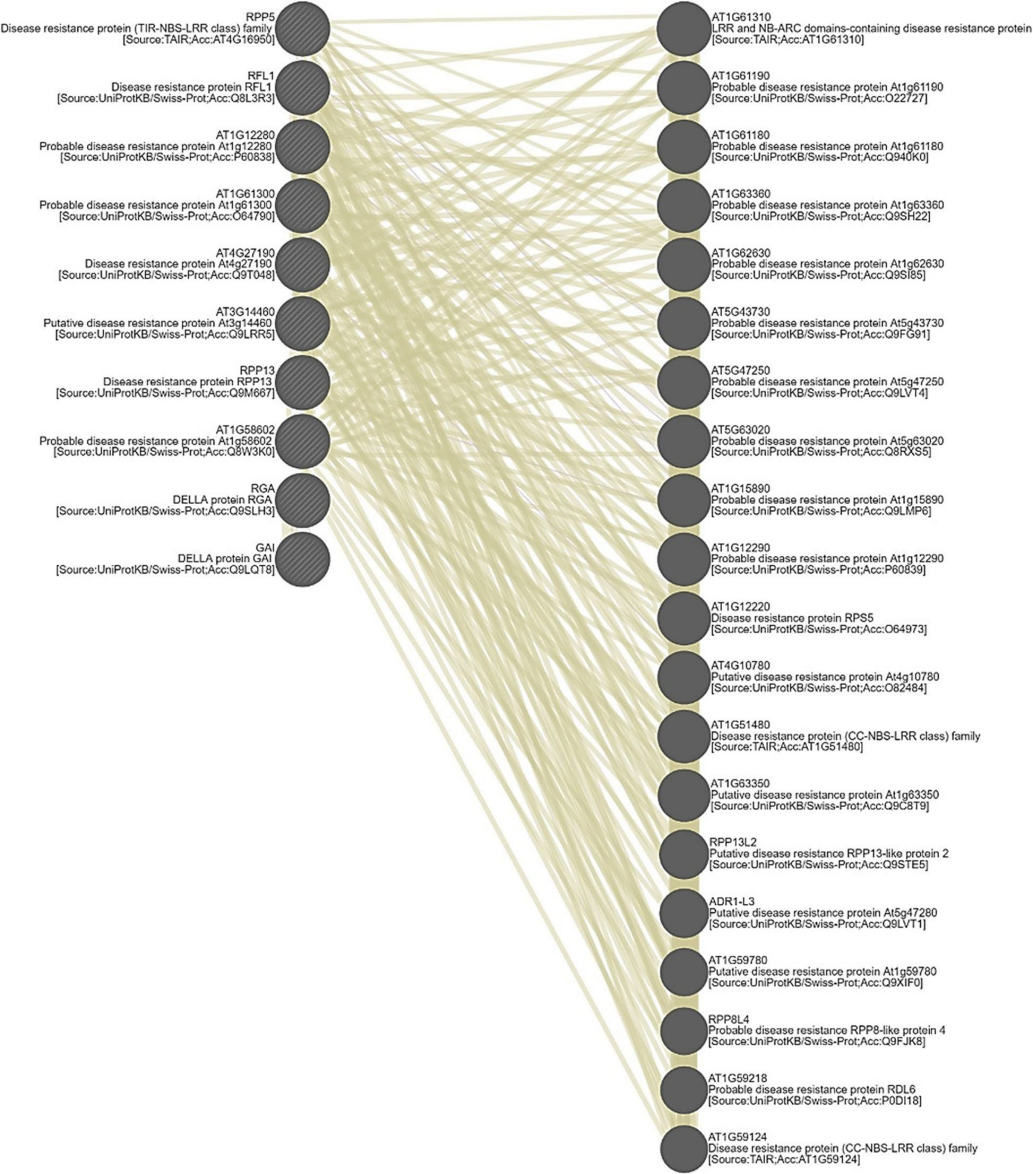
Predicted gene network for defense responsive genes in Arabidopsis genome generated using GeneMANIA platform

A large number of defensive genes were observed in contigs from the garlic transcriptome. These contigs were reported to have resistant and responsive properties against salt stress, high heat, viruses, bacteria, fungi, and light (Table 2). Defense response proteins revealed different conserved domains, such as RGA3, NBS-LRR, TIR-NBS-LRR, LRR, NBS-ARC, and CC-NBS-LRR. Furthermore, some other disease-resistant proteins (DRPs) were also recorded. Although their specific defense response activity was not found, defense-responsive proteins might play a major role in plant genetics. Among these proteins, two proteins (contigs: 68,405, 96,757) contain LLR and NB-ARC domains, followed by six other proteins (contigs: 4196, 43,673, 44,450, 102,622, 104,515, 119,410) with NBS-LRR domains and one TIR-NBS-LRR domain-based protein (contigs: 114,891). Moreover, nineteen disease-resistant proteins were also detected with the CC-NBS-LRR domain. Among DRPs, seven RPP13-like protein 1 proteins, seventeen Dirigent-like proteins, six At3g14460-like proteins, nine RPS2-like proteins, nine At1g12280-like proteins, eight At1g58602 isoform X3-like proteins, four RGA1-like proteins, five RGA2-like proteins, and a TMV N-like protein were also observed. Transgenic technology is the most important tool to deploy resistance genes and their ability in different plant species to promote or acquire resistance against numerous environmental stresses. GeneMANIA predicted a total of 30 functional partners of defense-responsive genes in the model plant A. thaliana (Fig. 7). Generated protein–protein interaction networks of defense-responsive genes in *A. thaliana* reveal that the regulatory partner of defense-responsive genes (Table 2) plays an important role in plant defense mechanisms.

**Table 2:** Predicted partners of defense responsive gene

Gene	Description	Rank
AT1G61300	Probable disease resistance protein At1g61300 [Source:UniProtKB/SwissProt;Acc:O64790]	N/A
AT1G58602	Probable disease resistance protein At1g58602 [Source:UniProtKB/SwissProt;Acc:Q8W3K0]	N/A
AT1G12280	Probable disease resistance protein At1g12280 [Source:UniProtKB/SwissProt;Acc:P60838]	N/A
RFL1	Disease resistance protein RFL1 [Source:UniProtKB/Swiss-Prot;Acc: Q8L3R3]	N/A
AT4G27190	Disease resistance protein At4g27190 [Source:UniProtKB/Swiss-Prot; Acc:Q9T048]	N/A
RPP13	Disease resistance protein RPP13 [Source:UniProtKB/Swiss-Prot;Acc: Q9M667]	N/A
AT3G14460	Putative disease resistance protein At3g14460 [Source:UniProtKB/SwissProt;Acc:Q9LRR5]	N/A
RGA	DELLA protein RGA [Source:UniProtKB/Swiss-Prot;Acc:Q9SLH3]	N/A
GAI	DELLA protein GAI [Source:UniProtKB/Swiss-Prot;Acc:Q9LQT8]	N/A
RPP5	Disease resistance protein (TIR-NBS-LRR class) family [Source:TAIR; Acc:AT4G16950]	N/A
AT1G61310	LRR and NB-ARC domains-containing disease resistance protein [Source: TAIR; Acc:AT1G61310]	1
AT1G61190	Probable disease resistance protein At1g61190 [Source:UniProtKB/SwissProt;Acc:O22727]	2
AT1G61180	Probable disease resistance protein At1g61180 [Source:UniProtKB/SwissProt;Acc:Q940K0]	3
AT1G63360	Probable disease resistance protein At1g63360 [Source:UniProtKB/SwissProt;Acc:Q9SH22]	4
AT1G62630	Probable disease resistance protein At1g62630 [Source:UniProtKB/SwissProt;Acc:Q9SI85]	5
AT5G43730	Probable disease resistance protein At5g43730 [Source:UniProtKB/SwissProt;Acc:Q9FG91]	6
AT5G47250	Probable disease resistance protein At5g47250 [Source:UniProtKB/SwissProt;Acc:Q9LVT4]	7
AT5G63020	Probable disease resistance protein At5g63020 [Source:UniProtKB/SwissProt;Acc:Q8RXS5]	8
AT1G15890	Probable disease resistance protein At1g15890 [Source:UniProtKB/Swiss-Prot;Acc:Q9LMP6]	9
AT1G122900	Probable disease resistance protein At1g12290 [Source:UniProtKB/Swiss-Prot;Acc:P60839]	10
AT1G12220	Disease resistance protein RPS5 [Source:UniProtKB/Swiss-Prot;Acc:O64973]	11
AT4G10780	Putative disease resistance protein At4g10780 [Source:UniProtKB/Swiss-Prot;Acc:O82484]	12
AT1G51480	Disease resistance protein (CC-NBS-LRR class) family [Source: TAIR; Acc:AT1G51480]	13
AT1G63350	Putative disease resistance protein At1g63350 [Source:UniProtKB/Swiss-Prot;Acc:Q9C8T9]	14
RPP13L2	Putative disease resistance RPP13-like protein 2 [Source:UniProtKB/Swiss-Prot; Acc: Q9STE5]	15
ADR1-L3	Putative disease resistance protein At5g47280 [Source:UniProtKB/Swiss- Prot;Acc:Q9LVT1]	16
AT1G59780	Putative disease resistance protein At1g59780 [Source:UniProtKB/Swiss- Prot;Acc:Q9XIF0]	17
RPP8L4	Probable disease resistance RPP8-like protein 4 [Source:UniProtKB/ Swiss-Prot;Acc:Q9FJK8]	18
AT1G59218	Probable disease resistance protein RDL6 [Source:UniProtKB/Swiss- Prot;Acc:P0DI18]	19
AT1G59124	Disease resistance protein (CC-NBS-LRR class) family [Source:TAIR; Acc:AT1G59124]	20

### Comparative analysis of orthologous gene family

**Fig. 8:**
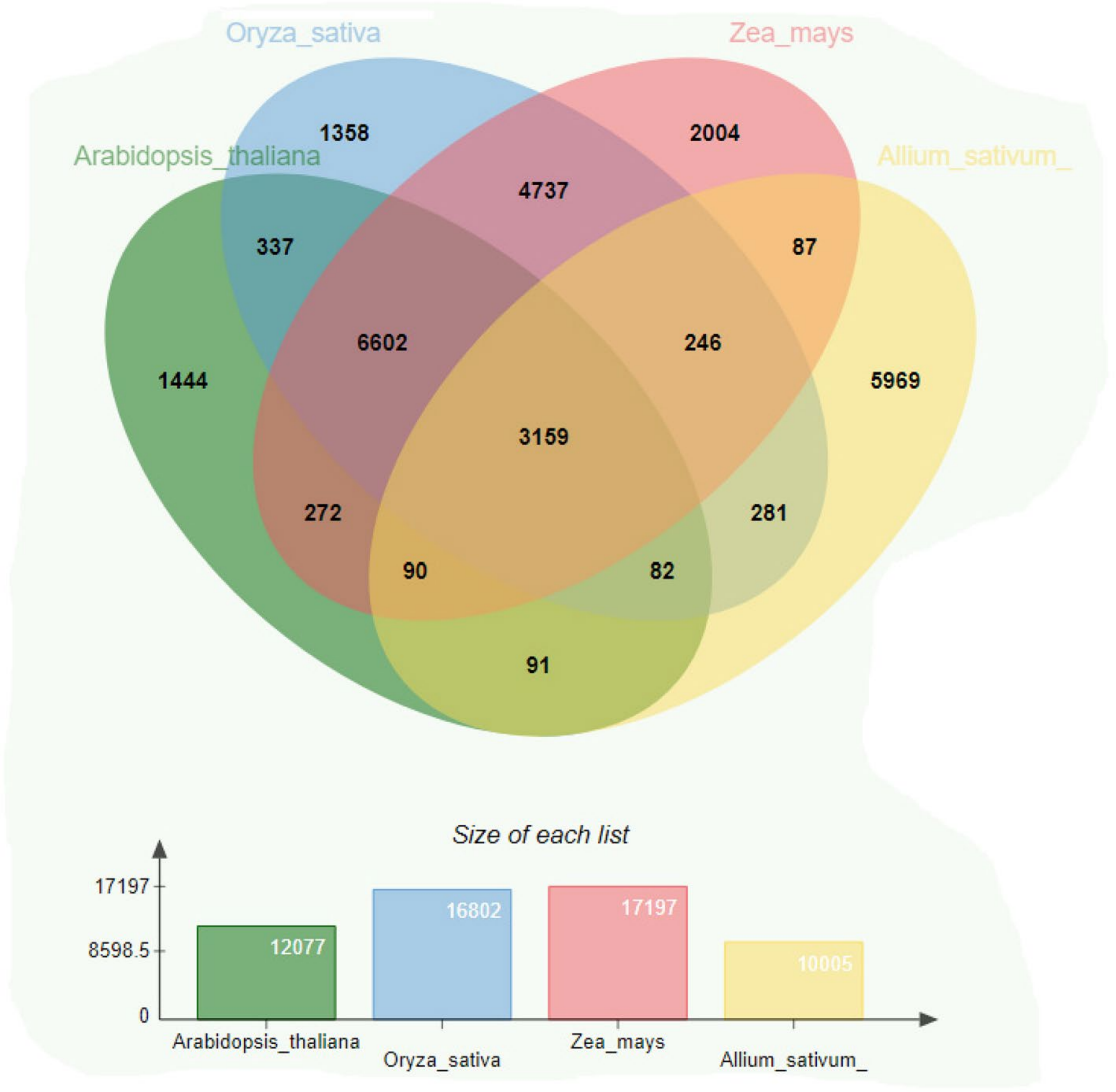
Venn diagram displays the distribution of shared orthologous clusters among the four species (*A. sativum*, *A. thaliana*, *O. sativa*, and *Z. mays*)

The comparison of orthologous groups of the *Allium sativum* transcriptome was compared with *Arabidopsis thaliana*, *O. sativa*, and *Zea mays*. Orthology analysis was performed among (*A. sativum*), 12,077 (*A. thaliana*), 16,802 (*O. sativa*), and 17,197 (*Z. mays*) unigenes using orthoVenn (Fig. 8). A total of 10,005 orthologous groups were identified, of which 5969 were discovered in *A. sativum*, 3159 in *A. thaliana*, 246 in *O. sativa*, and 87 in *Z. mays*. Functional analysis showed that the distribution patterns of the GO categories were more similar between *A. sativum* and *A. thaliana* than between *A. sativum* and *O. sativa* than between *A. sativum* and *Z. mays*. This indicated that the transcriptomic profile of *A. sativum* is similar to that of *A. thaliana* than to that of *O. sativa*, which is consistent with their species classification. Some GO slim terms were specifically assigned to *A. sativum* and *A. thaliana*, such as “defense response (GO:0,006,952)” and “metabolic process” (GO:0,008,152) in the biological process category, “signal transducer activity (GO:0,004,871),” and “transporter activity (GO:0,005,215) in the molecular function category.

### Molecular markers identification based on the de novo assembled transcriptome

**Fig. 9:**
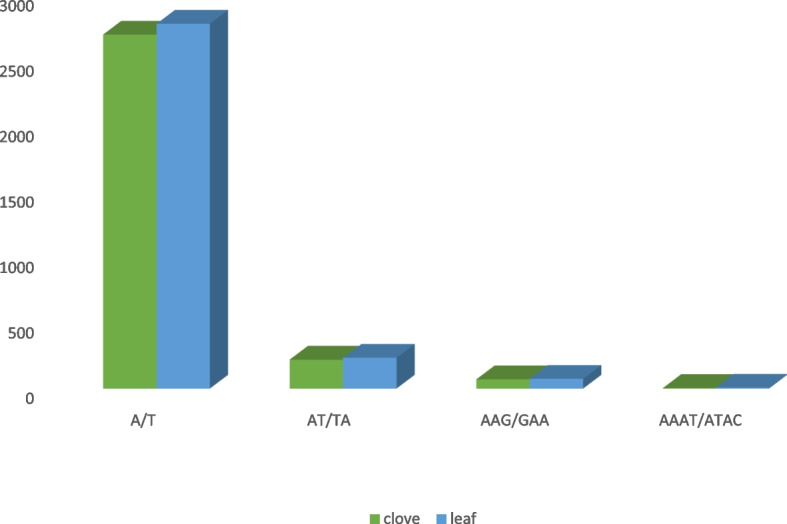
Bar graph showing unique SSR motif in garlic transcriptome

To build a genomic resource for further genetic improvement of garlic, simple sequence repeat markers were identified in the contigs of clove and leaf. A total of 8393 putative SSRs were obtained from the contigs of garlic clove. A total of 564 SSRs were involved in compound formation, and 746 transcripts contained more than 1 SSR. Furthermore, 7403 SSRs were obtained from leaf contigs (Table 3). A total of 128 unique motifs were found, and of these, mononucleotide repeats A and T were highly abundant, i.e., 2696 and 2779, respectively. However, in the case of di repeats, AT and TA repeats were found the maximum number of times, i.e., 219 and 233, respectively, while AAG and GAA tri repeats were found 71 and 75 times, respectively, and while AAAT and ATAC tetra repeats were found 4 and 7 times, respectively (Fig. 9).

**Table 3:** SSRs markers identified from the garlic transcriptome

	Clove	Leaf
Total number of sequences examined	131,305	108,668
Total size of examined sequences (bp)	70,854,269	62,021,110
Total number of identified SSRs	8393	7403
Number of SSR containing sequences	7460	6661
Number of sequences containing more than 1 SSR	746	614
Number of SSRs present in compound formation	564	420
Mono	5743	5183
Di	1315	1014
Tri	1244	1111
Tetra	73	76
Penta	9	10
Hexa	9	9

## Discussion

For several nonmodel species without available genomic reference information, transcriptome sequencing is an effective and alternative method to gain insight into the information content of a genome. To date, several studies have published *Allium sativum* L. transcriptomes by sequencing cDNA libraries and Illumina sequencing [5, 13]. Transcriptome assembly for various organisms, including *Cleome spinosa* and *Cleome gynandra* [6], *Dodonaea viscosa* [35], and 12 Citrus species [30], has already been reported using the same approach.

In this study, we constructed a transcriptome of *Allium sativum* by assembling approximately 16 Gb RNA-Seq PE read data. The draft transcriptome consists of 131,305 contigs, of which 137,307 were annotated using the OmicsBox pipeline. Due to a lack of genomic resources, the proportions of contigs to known proteins in GenBank were considered a very useful metric. In total, 42% of contigs were matched to known proteins in public databases. This implies that our Illumina paired-end sequencing generated a considerable portion of the *Allium sativum* genes. Taking into account all BLAST hits in the NCBI NR protein database, the top ranked species with the most matched annotations were *Asparagus officinalis* (~ 30%), followed by *Arabidopsis thaliana* (~ 10%), *Oryza sativa* (~ 4%), and *Zea mays* (~ 3%). Protein kinase-like domain (IPR011009), leucine-rich repeat domain superfamily (IPR032675), zinc finger (IPR013083), and cytochrome P450 superfamily (IPR036396) protein domains were more prevalent in the garlic transcriptome. The present study revealed more pathways related to cellular processes, and environmental information processing was also well represented by the contigs from *Allium sativum*. Most of these assignments were based on marginal BLAST hits near the significance threshold and often involved low-complexity sequences, which are prone to producing false-positive hits [26]. There are still contigs without significant matches to the existing databases, which could reflect either novel, specific genes of *Allium sativum* or noncoding RNAs or fragments of longer RNAs.

Further study with GO analysis revealed that *Allium sativum* plant genes are involved in many biological processes, and many genes were classified as “metabolic process,” “organonitrogen compound biosynthesis,” and “catalytic activity,” which in turn suggests a large diversity of enzymes involved in various syntheses of metabolites. Pathway-based analysis is helpful for understanding the biological functions and interactions of genes.

Plants have an innate immune system to defend themselves against pathogens by a number of mechanisms, such as hypersensitive response (HR), induction of genes encoding PR and/or induced biosynthesis of secondary metabolites. In plants, HR is a form of programmed cell death (PCD) at the site of pathogen infection, which is closely related to active resistance.

Disease resistance genes (R genes) in plants frequently encode leucine-rich repeat (NBS-LRR) proteins, and the leucine-rich repeat (LRR) domain present in RLK interacts with other proteins, leading to a signaling response [13]. Thus, RLK gene family proteins play an important role in pathogen recognition by signaling pathways that lead plants to activate their plant defense mechanism and provide resistance against disease [20, 27, 31]. In the present study, we identified 291 disease resistance contigs in the garlic transcriptome. CC-NBS-LRR and TIR-NBS-LRR protein domains were observed frequently, which is quite analogous to the R genes found in *C. arabica* and *C. canephora* [25]. NBS-LRR (nucleotide-binding site leucine-rich repeat) proteins involved in the activation of kinases play an important role in plant defense mechanisms against pathogen invasion [22]. The nonspecific transcription factor family were classified into families such as C2H2, WD40-like, and MYB-HB-like [7]. Generally, mitogen-activated protein kinase (MAPK) cascades are initiated by stimulated receptors. After a series of cascade reactions, activated MAPKs phosphorylate their substrates, most of which are enzymes and transcription factors, thereby triggering downstream responses [23]. These conserved domain protein sequences represent major R-gene classes of plant resistance genes [14]. All other defensive genes showed similarity with *Arabidopsis thaliana*, *Citrus sinensis*, *Zea mays*, etc. Similar results were reported in *Arabidopsis thaliana* and *Dimocarpous longan* [11, 26].

Transcription plays an important role in the defense mechanism of plants against biotic and abiotic stresses and signal transduction during pathogen invasion [3, 4]. WRKY TFs, as substrates of MAPKs, can be regulated by MAPKs at the transcriptional and/or posttranslational levels [16, 17, 27]. WRKY TFs can activate downstream disease response genes or hormone pathway-related genes to protect against pathogen infection [18, 29]. In our BLAST results against the plant transcription factor database, we found maximum transcript hits with CCHC(Zn), WD40, BED-type (Zn), C3H, NAC, C2H2, WRKY, and AUX-IAA transcription factors. The C2H2 transcription factor with a high number of contigs was identified in the present study. These genes play an important role in independent metal binding and are capable of binding with Zn elements [10], and a study also report that the C2H2 transcription factor plays an important role in the defense response and various physiological processes [33]. In addition, many studies have shown that TFs that contain the NAC domain play pivotal roles in the regulation of transcriptional reprogramming associated with plant stress responses, such as abiotic stress responses and pathogen defense [24].

The generic region of simple sequence repeats (SSR) can be used for ecological studies, linkage mapping functional domain markers, quantitative trait loci (QTL) exploration, evolutionary studies, comparative genomics, and genetic diversity. To date, in garlic, very little information on SSR markers from generic and genomic regions is available [15]. In a previous study, 17,374 SSR markers were identified in snow mountain garlic [21]. In the present study, we identified 8393 and 7403 generic SSR markers in the garlic clove and leaf transcriptome, respectively, and designed sets of primers for defense-responsive genes. The contig information obtained in the present study has great significance in the functional genomics of the non-model plant *Allium sativum* L. for defensive genes and the interaction of these genes. Furthermore, this information can also be used to understand plant pathogen interaction mechanisms. The identified defensive gene SSR motif and designed primes add useful information in germplasm and screening for different ecospecies. The identified defensive genes and TFs can be used for the development of biotic stress and abiotic tolerant transgenic plants of commercial importance.

## Conclusions

The present study provides insight into the transcriptomics and characterization of defensive genes of Garlic (*A. sativum*) by generating > 43 million paired-end reads (125 × 2) using the Illumina HiSeq 2000 platform, which were assembled into 239,973 transcripts. This study characterized the likely coding genes present in these assemblies and further identified plant transcription factors and defensive genes in clove and leaf samples of garlic. These defensive genes were functionally characterized using BLAST, Gene Ontology terms, KEGG pathways, Pfam domains, and families. The study proposed the genes of the plant pathogen interaction pathway and their protein–protein interaction network. It also identified some important candidate genes that may play important roles in plant defense and plant immunity, such as RGA1, RHA3, RNA-dependent RNA polymerase gene, LLR receptor serine threonine-kinase, ANP1-like, NAC, PR%, NBS-LRR, MYB, bHLH, RPP13-like protein 1, At3g14460-like protein, RPS2-like protein, At1g12280-like protein, At1g58602 isoform X3-like protein, and TMV N. It is expected that this resource would contribute substantially to understanding the plant pathogen interaction in this plant. Using sequencing data, this study further identified molecular markers, such as simple sequence repeat markers, and designed identified defensive gene SSR marker primers, which present a substantial resource of this plant for genetic study and further crop improvement. In brief, this study provides a substantial genetic resource for the Garlic cultivar Yamuna safed-3 (G-282), which has opened up new avenues for further molecular interventions.

## Data Availability

The SRA of garlic have been deposited in NCBI under accession number SRX8876914 and SRX8862808.

## Abbreviations

GO: Gene Ontology
NCBI: National Center for Biotechnology Information
Nr: Non-redundant protein database
COG: Cluster orthologous gene database
KEGG: Kyto Encyclopedia Gene and Genome Database
RAG: Resistance gene analogs
LRR: Leucine-rich repeats
NBS-LRR: Nucleotide-binding site-leucine-rich repeat
bZIP: Basic region-leucine zipper
SSR: Simple sequence repeat
MISA: Microsatellites identification tool
BLAST: Basic Local Alignment Search Tool
HR: Hypersensitive response
TF: Transcription factor
MAPK: Mitogen-activated protein kinase
